# The shape of the transverse arch in high heels while standing

**DOI:** 10.1371/journal.pone.0233958

**Published:** 2020-06-08

**Authors:** Hala Zeidan, Mirei Kawagoe, Yuu Kajiwara, Keiko Harada, Yurika Nishida, Keisuke Yamada, Rika Kawabe, Junpei Yokota, Chiaki Yamashiro, Yu Odake, Masakatsu Takeda, Naoki Doi, Kaho Negoro, Natsuki Matsumura, Tappei Morino, Clemence Kiho Bourgeois Yoshioka, Chang Yu Chen, Tomoki Aoyama

**Affiliations:** 1 Department of Physical Therapy, Human Health Sciences, Graduate School of Medicine, Kyoto University, Kyoto, Japan; 2 Department of Physical Therapy, Faculty of Health Science, Kio University, Koryo, Japan; University of Glasgow, UNITED KINGDOM

## Abstract

**Introduction:**

High heeled shoes have long been worn in society and they are known to cause biomechanical imbalances to not only the foot, but the whole musculoskeletal system. This study aims to show the detailed changes that happen to the shape of the transverse arch of the foot in high heels, using two different inclination degrees.

**Methods:**

68 women participated in this study. Two custom-made high heels were made with inclinations of 15 degrees and 30 degrees (cm). A weight-bearing ultrasound was used to assess the coronal view of the transverse arch in standing. ANOVA and Tuckey tests were used to compare the results between 0 degrees, 15 degrees and 30 degrees inclinations.

**Results:**

The transverse arch height was slightly increased as the heel height increased (0DI-15DI: p = 0.5852 / 15DI-30DI: p = 0.395 / 0DI-30DI: p = 0.0593). The transverse arch length (0DI-15DI: p = 0.0486 / 15DI-30DI: p = 0.0004 / 0DI-30DI: p = 0.1105) and the area under the metatarsal heads (0DI-15DI: p = 0.0422 / 15DI-30DI: p = 0.0180 / 0DI-30DI: p = 0.9463) significantly decreased as the heel height increased.

**Discussion:**

The main changes were viewed in the 30 degrees inclinations compared to 0 degrees inclination. When the toes are dorsiflexed in high heels, it stimulates the Windlass mechanism which in turn stiffens the plantar fascia and adducts the metatarsal heads, while the soft tissues shrink in response to loads.

**Conclusion:**

High heels affected the shape of the transverse arch even in short term standing, and these effects increased as the height of the heel increased.

## Introduction

High heeled footwear (HH) are shoes that are higher under the heels than under the other parts of the shoe [1, 2] and they vary in shape and height [1–3]. They have been worn for centuries and are still worn nowadays, mainly by women, for work and for beauty and fashion purposes [1, 4–6]. Wearing HH interferes with natural foot biomechanics [1, 2] and affects the musculoskeletal system [2, 7], and these changes are said to be compensatory mechanisms that the body takes to adapt to its environment [5] and are worsened in elderly women [5, 6].

Wearing HH for a long term is said to lead to chronic foot pain and deformities [7].

In the literature, HH are said to cause calluses [7], postural changes of the lower limbs and spine; such as lumbar hyperlordosis [1, 2, 4] and valgus knees [1, 4]; tightening and shortening of the soleus which decreases the ankle’s range of motion [7]; overuse of the muscles and increased energy expenditure [4] which in consequence leads to muscle fatigue [3, 4]; instability of the ankle, which is a leading cause of injuries, sprains and falls in HH [3]; difficulty to maintain body balance [1, 5] due to ankle instability and the forward shift of the center of gravity [1]; knee osteoarthritis [2, 4]; deformities in the toes, such as hallux valgus [3, 7] which in its turn leads to forefoot pain [8]. HH also change gait patterns and plantar pressure distribution under the foot because of heel elevation [3, 8], with increased pressure on the forefoot [1, 8, 9] and transverse arch of the foot [6]. Furthermore, past studies reported more pressure on the first [2, 8] and second metatarsal heads [8]. The gravity of HH effects depend on the height [1, 3, 4], the type [1] and the broadness of the heel’s base [3], the age when one starts wearing HH (worse at younger age) [7, 9], the duration [6] and the experience [2] of wearing HH.

The transverse arch is one of the three arches of the foot [10, 11] which respond to weight when walking and provide shock absorption during gait [11, 12]. The transverse arch is located in the forefoot and lays under the metatarsal heads [10, 12, 13]. Its role is to absorb forces and help in forward propulsion during gait. In HH, loads are shifted to the forefoot [4, 7], and these loads increased as the height of the HH increases. Therefore; we decided to study the impact that HH has on the shape of the transverse arch.

This study aims to describe the changes that happen to the shape of the transverse arch when standing with HH on. We used an ultrasound machine that allows to view the coronal plane of the transverse arch and custom-made high heels. We measured the transverse arch height (TAH), heights of the metatarsal heads, sesamoid rotation angle (SRA), transverse arch length (TAL), length between the metatarsal heads, total area under the metatarsal heads and the areas between the metatarsal heads; in three inclination angles: flat (0°), moderately high heels (15°), and high heels (30°). We hypothesize that the arch will drop and widen while the area underneath the metatarsal heads will decrease, and that this change increases as the height of the heel increases.

## Methods

### Participants

During a health care event for women in Aichi prefecture–Japan, in 2016, 90 adult women voluntarily participated in taking their foot measurements. The purpose of the study was explained to them, and signed consents were obtained from each participant before the measurements. Exclusion criteria were: history of lower leg surgery or fracture of the foot and ankle, disc herniations, neurological or congenital disorders, diabetes and pregnancy. The current study was performed in accordance with the current local guidelines and the Declaration of Helsinki and approved by the Ethical Committee of Kyoto University Graduate School and Faculty of Medicine (approval number: R0450).

### Measurement equipment

**Fig 1:**
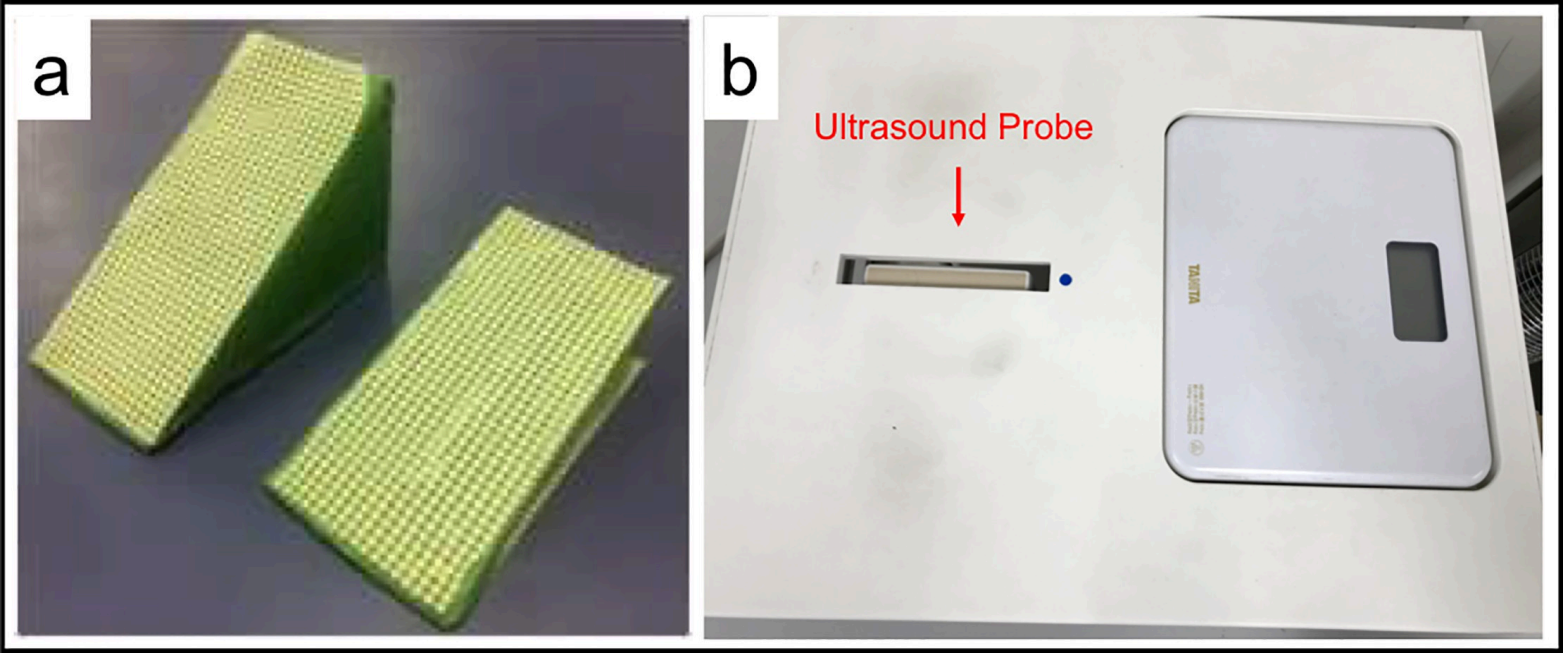
Measurement equipment. a) two custom-made high heels of 15 degrees inclination and 30 degrees inclination; b) WPUID containing an ultrasound probe and a weight scale, allows to take coronal views of the transverse arch of the foot.

Two custom-made HH were prepared for this study. These two HH were made with flat hard mattress board and slip resistant sheets were attached on the inclined plane and basal plane of each HH (Fig 1A). These HH had two inclination degrees: 15 and 30 degrees. These inclination degrees corresponded to heel heights (in cm) depended on the participants’ foot size (in cm). Seven centimeters (about 2.76 inch) is the most common high heel height for users and 23.5 cm (about 9.25 inch) to 24 cm (about 9.06 inch) is the most common foot size for Asian women. A 30 degrees inclined heel indicated a 7 cm (about 2.76 inch) heel height for participants with a 24 cm (about 9.06 inch) foot size when standing on the modified heel with midfoot and rearfoot. As this was an early-stage study in HH research, we could not customize the modified heels for each person in the experimental setup, therefore, the inclination angles (in degrees) were standardized. The time of measurement for each participant was limited considering fatigue from repetitive measurements.

A weight-bearing plantar ultrasound imaging device (WPUID) (Fig 1B) was made to view the coronal plane of the transverse arch in weight-bearing condition. This device was described and used in previous studies [1, 3, 13]. It is a rectangular shaped box which has an opening allowing to protrude an ultrasound probe (EUP—L53L; Hitachi Aloka Medical, Tokyo, Japan) from inside the box, upside-down. The ultrasound machine Noblus 128 (Hitachi Aloka Medical) was used to take images of the arch. This device also had a weight scale inserted next to the ultrasound probe allowing to control weight shifts during measurements. This device was previously used in other studies [10, 13–15].

### Measurement protocol

First, participants were seated and set their dominant foot on the solid gel of the WPUID and the other foot on the digital weight scale; then, the examiner fixed the position of the participants’ sesamoid bones and 5th metatarsal head (5MTH) on the solid gel area. Second, the participants stood on the WPUID, holding their foot on the solid gel area and the other foot on the digital scale until the digital scale showed almost half of their weight. Then, 0 degrees inclination (0DI) condition’s ultrasound images were taken. Third, the examiner placed the 15 degrees inclined heels under their midfoot to rearfoot holding both feet stable and took ultrasound images in the 15 degrees inclination (15DI) condition when the digital scale of the other side of the foot indicated half of their weight. Then, ultrasound images in the 30 degrees inclination (30DI) condition were taken in the same manner. B-mode ultrasound images were taken at a frequency of 9.0 MHz once by one researcher for each condition, considering the participants’ fatigue. A total of 3 ultrasound images were taken per foot.

### Ultrasound image analysis

**Fig 2:**
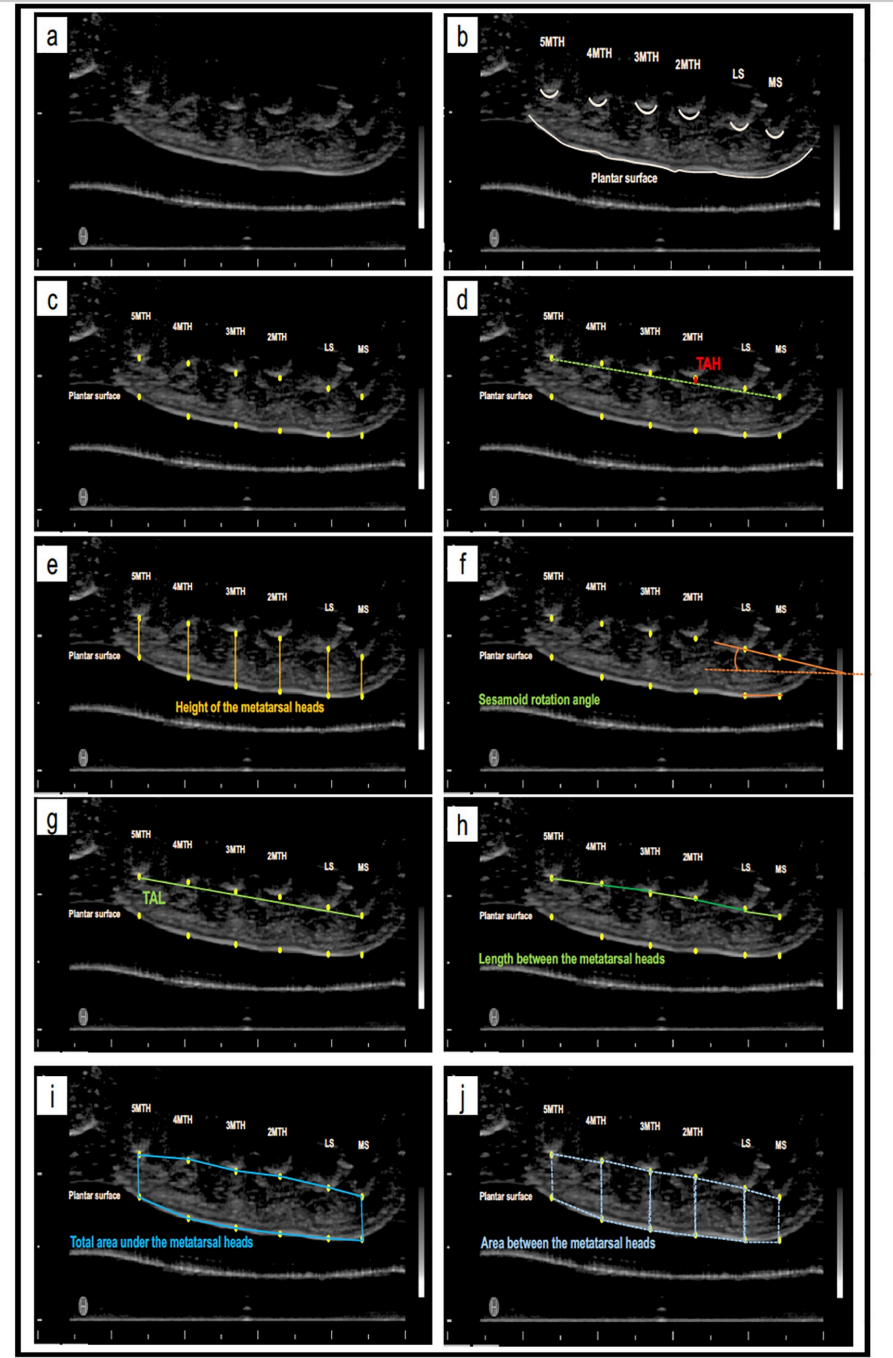
Ultrasound image analysis. a) ultrasound image of the transverse arch; c) marking the lowest points of MS, LS, 2MTH, 3MTH, 4MTH, 5MTH and their plantar surface projections; d) TAH is red line; e) the height of the metatarsal heads in orange lines; f) SRA in orange angle; g) TAL in green line; h) the length between each metatarsal head in green lines; i) the total area under the metatarsal heads marked by blue lines; j) the area between each two metatarsal heads marked by dotted blue lines.

After transferring all ultrasound images to a computer, they were analyzed using ImageJ software (ImageJ; National Institute of Health, Maryland, USA) (Fig 2A, Fig 2B). Twelve points were marked and their (x, y) coordinates were used in the analysis on an Excel spreadsheet. These points were: the lowest point the medial sesamoid (MS), lateral sesamoid (LS), second to fifth metatarsal head (MTH) bones, and 6 points on the plantar surface of the skin (Fig 2C). The plantar points are the vertical projection of each bone on the adjacent plantar skin. The X-axis were standardized to confirm the vertical points. TAH was defined as the line starting from 2MTH and perpendicular to the line between MS and 5MTH (Fig 2D). The height of the metatarsal heads was measured by subtracting the PS from the metatarsal head (e.g., 2MTH height would be [2MTH] = 2MT-2PS; Fig 2E). SRA is the angle between the line formed by MS and LS points and the line formed by the plantar projections of MS and LS (Fig 2F). TAL was defined as the length between MS and 5MTH (Fig 2G). TAH ratio was defined as the percentage of transverse arch height divided by TAL. The length between each metatarsal head was also analyzed (Fig 2H). The total area under the transverse arch was defined using each of the twelve points (Fig 2I). The area between the metatarsal heads was measured using two metatarsal heads and their adjacent plantar projections (Fig 2J). The points used for the measurement are shared between the measurements themselves (for example: TAH is calculated using the coordinates of 2MTH and the TAL (using the coordinates of MS and 5MTH)), making the measurements related to each other. These measurements were previously used in other studies [10, 13–15].

### Statistical analysis

Statistical analysis was done using JMP Pro 14 (version 12.2.0, SAS Institute Inc., Cary, NC, USA). One-way ANOVA was used to compare the parameters between the three conditions (0DI, 15DI, 30DI) and Tukey test was used as a post-hoc test. The p-value was set at 0.05. All results are displayed as mean ± standard deviation.

## Results

**Fig 3:**
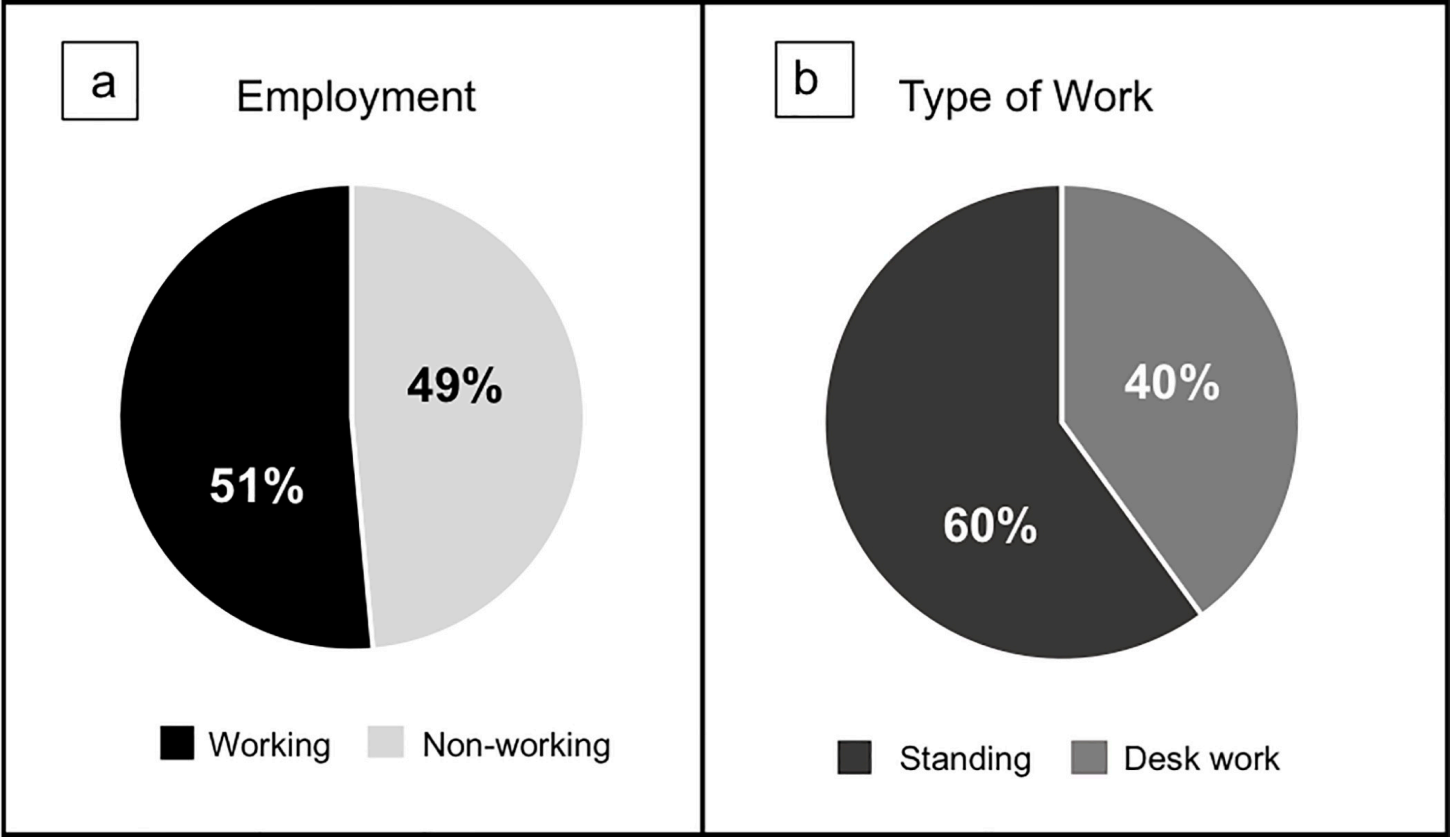
Employment and type of work. a) percentages of women working and nonworking; b) percentage of the type of work of working women.

The total number of the participants was 90 adult women and after applying the exclusion criteria and excluding the ultrasound images that were not clear for analysis, 68 women (68 dominant feet) were left for the analysis of this study. The demographic data of the participants are represented in Table 1. Fig 3 represents the percentages of the working and non-working women (Fig 3A), and the type of work (Fig 3B).

**Table 1:** Demographic data of the participants (n = 68).

	Mean ± SD
Age (years)	38.29 ± 8.20
Body Height (cm)	157.76 ± 5.47
Body Weight (kg)	52.59 ± 9.29
Body Mass Index (kg/m2)	21.12 ± 3.58
HVA (degrees)	11.43 ± 7.10
Foot Length (cm)	22.74 ± 1.00
Foot width (cm)	9.03 ± 0.50

**Fig 4:**
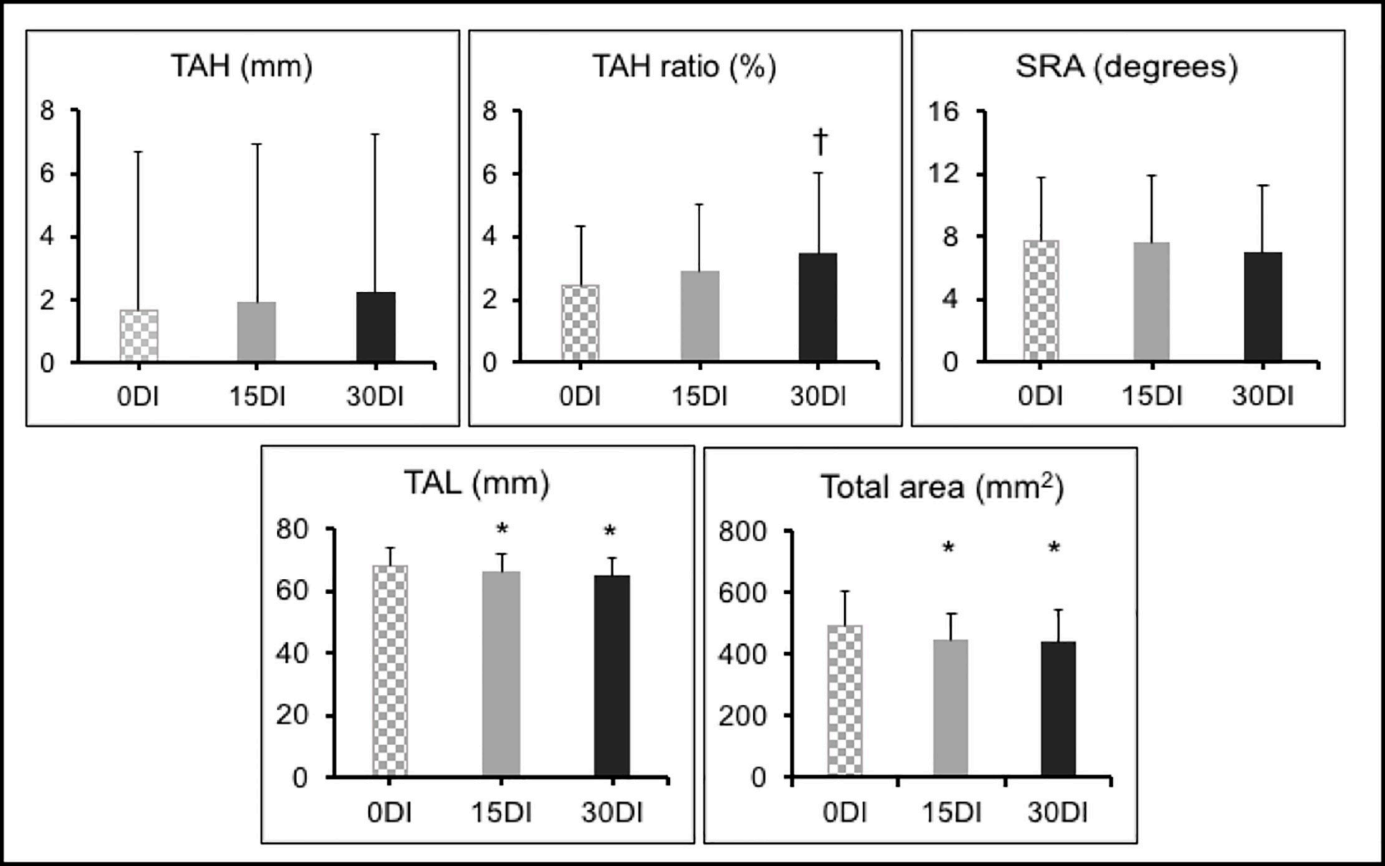
TAH, TAH ratio, SRA, TAL and total area. *significant compared to 0 degrees; † significant compared to 15 degrees. TAH transverse arch height; SRA sesamoid rotation angle; TAL transverse arch length; mm millimeters; mm^2^ square millimeters.

The results of TAH, TAH ratio, SRA, TAL and total area in the three measurement conditions are represented in Fig 4. The TAH increased without significance between 0DI (1.67 ± 1.27) and 15DI (1.92 ± 1.41) (p = 0.5852), between 15DI and 30DI (2.24 ± 1.69) (p = 0.395) and between 0DI and 30DI (p = 0.0593). TAH ratio increased without significance between 0DI (2.45 ± 1.87) and 15DI (2.91 ± 2.13) (p = 0.2275) and between 15DI and 30DI (3.45 ± 2.57) (p = 0.1522), but there was significant increase between 15DI and 30DI (p = 0.0087). SRA decreased without significance between 0DI (7.77 ± 4.07) and 15DI (7.54 ± 4.36) (p = 0.7519), between 15DI and 30DI (6.90 ± 4.37) (p = 0.3877) and between 0DI and 30DI (p = 0.2385). TAL decreased significantly between 0DI (68.21 ± 5.53) and 15DI (66.28 ± 5.36) (p = 0.0486) and between 0DI and 30DI (64.72 ± 6.14) (p = 0.0004), but there was no significance decrease between 15DI and 30DI (p = 0.1105). Total area under the metatarsal heads decreased significantly between 0DI (489.63 ± 115.97) and 15DI (446.69 ± 84.41) (p = 0.0422) and between 0DI and 30DI (441.10 ± 106.23) (p = 0.0180), but there was no significance decrease between 15DI and 30DI (p = 0.9463).

The results of the metatarsal head heights in the three measurement conditions are represented in Table 2. 2MTH height was significantly decreased in the 30DI condition compared to 0DI (p = 0.0021). 3MTH height was significantly decreased in the 30DI condition compared to 0DI (p = 0.0118). 4MTH height was significantly decreased in the 30DI condition compared to 0DI (p = 0.0036).

**Table 2:** MTH height.

	0DI	15DI	30DI
MS height (mm)	7.044 ± 1.87	6.71 ± 1.70	6.77 ± 1.63
LS height (mm)	8.59 ± 2.00	8.44 ± 2.06	8.06 ± 2.15
2MTH height (mm)	8.46 ± 2.13	8.06 ± 2.05	7.22 ± 2.14 *
3MTH height (mm)	9.11 ± 1.916	8.69 ± 1.79	8.14 ± 2.15 *
4MTH height (mm)	9.07 ± 1.91	8.72 ± 1.73	8.04 ± 1.85 *
5MTH height (mm)	7.27 ± 1.88	6.74 ± 1.56	6.81 ± 1.76

*significant compared to 0 degrees; † significant compared to 15 degrees. 0DI 0 degrees of inclination; 15DI 15 degrees of inclination; 30DI 30 degrees of inclination; MS medial sesamoid; LS lateral sesamoid; 2MTH second metatarsal head; 3MTH third metatarsal head; 4MTH forth metatarsal head; 5MTH fifth metatarsal head; mm millimeters.

The results of the length between the metatarsal heads in the three measurement conditions are represented in Table 3. Only the length between 4MTH and 5MTH was significantly decreased in 30DI compared to 0DI (p = 0.0260).

**Table 3:** Length between the metatarsal heads.

	0DI	15DI	30DI
MS~LS (mm)	14.97 ± 3.03	15.10 ± 3.50	14.55 ± 3.08
LS~2MTH (mm)	21.85 ± 5.21	19.93 ± 4.30	20.04 ± 5.18
2MTH~3MTH (mm)	18.55 ± 3.05	18.99 ± 4.10	18.11 ± 4.43
3MTH~4MTH (mm)	19.39 ± 3.71	19.43 ± 3.46	18.86 ± 3.68
4MTH~5MTH (mm)	21.77 ± 3.66	20.29 ± 3.83	20.00 ± 4.39 *

*significant compared to 0 degrees; † significant compared to 15 degrees. 0DI 0 degrees of inclination; 15DI 15 degrees of inclination; 30DI 30 degrees of inclination; MS medial sesamoid; LS lateral sesamoid; 2MTH second metatarsal head; 3MTH third metatarsal head; 4MTH forth metatarsal head; 5MTH fifth metatarsal head; mm millimeters.

The results of the area between the metatarsal heads and the plantar skin in the three measurement conditions are represented in Table 4. The area between LS and 2MTH decreased significantly in 15DI compared to 0DI (p = 0.0470) and in 30DI compared to 0DI (p = 0.0009). The area between 2MTH and 3MTH decreased significantly in 30DI compared to 0DI (p = 0.0295). The area between 3MTH and 4MTH decreased significantly in 30DI compared to 0DI (p = 0.0043). The area between 4MTH and 5MTH decreased significantly in 15DI compared to 0DI (p = 0.0136) and in 30DI compared to 0DI (p = 0.0003). The coordinates used in measuring the area between the MTH are related to the ones used in for MTH height and length between the MTH, making these results related to each other.

**Table 4:** Area between the metatarsal heads and the plantar skin.

	0DI	15DI	30DI
MS~LS (mm^2^)	83.80 ± 27.67	82.50 ± 32.82	77.76 ± 28.51
LS~2MTH (mm^2^)	131.64 ± 42.78	115.95 ± 34.91 *	107.46 ± 36.87 *
2MTH~3MTH (mm^2^)	115.00 ± 30.83	112.82 ± 34.70	99.35 ± 40.46 *
3MTH~4MTH (mm^2^)	124.44 ± 32.90	119.21 ± 29.02	107.56 ± 29.71 *
4MTH~5MTH (mm^2^)	126.34 ± 33.66	111.13 ± 28.37 *	105.14 ± 31.32 *

*significant compared to 0 degrees; † significant compared to 15 degrees. 0DI 0 degrees of inclination; 15DI 15 degrees of inclination; 30DI 30 degrees of inclination; MS medial sesamoid; LS lateral sesamoid; 2MTH second metatarsal head; 3MTH third metatarsal head; 4MTH forth metatarsal head; 5MTH fifth metatarsal head; mm^2^ square millimeters.

## Discussion

In this study, we aim to describe the changes that happen to the shape of the transverse arch of Japanese young women when standing at 0, 15 and 30 degrees of heel inclination; using a weight-bearing ultrasound device. The significant changes that our results showed are 1) significantly decreased TAL and area under the metatarsal heads as heel inclination increased, 2) significantly decreased heights of the 2MTH, 3MTH and 4MTH in 30DI compared to 0DI, 3) significantly decreased area between LS and 2MTH, 2MTH and 3MTH, 3MTH and 4MTH, 4MTH and 5MTH in 30DI compared to 0DI. The main significantly different results were between 0DI and 30DI. However even in 15DI, the TAL significantly shortened and the areas between LS and 2MTH and 4MTH and 5MTH significantly decreased, compared to 0DI, indicating that even lower HH height affects the shape of the transverse arch. In both heights (15DI and 30DI), the space between LS and 2MTH and the one between 4MTH and 5MTH seem to be the most affected.

**Fig 5:**
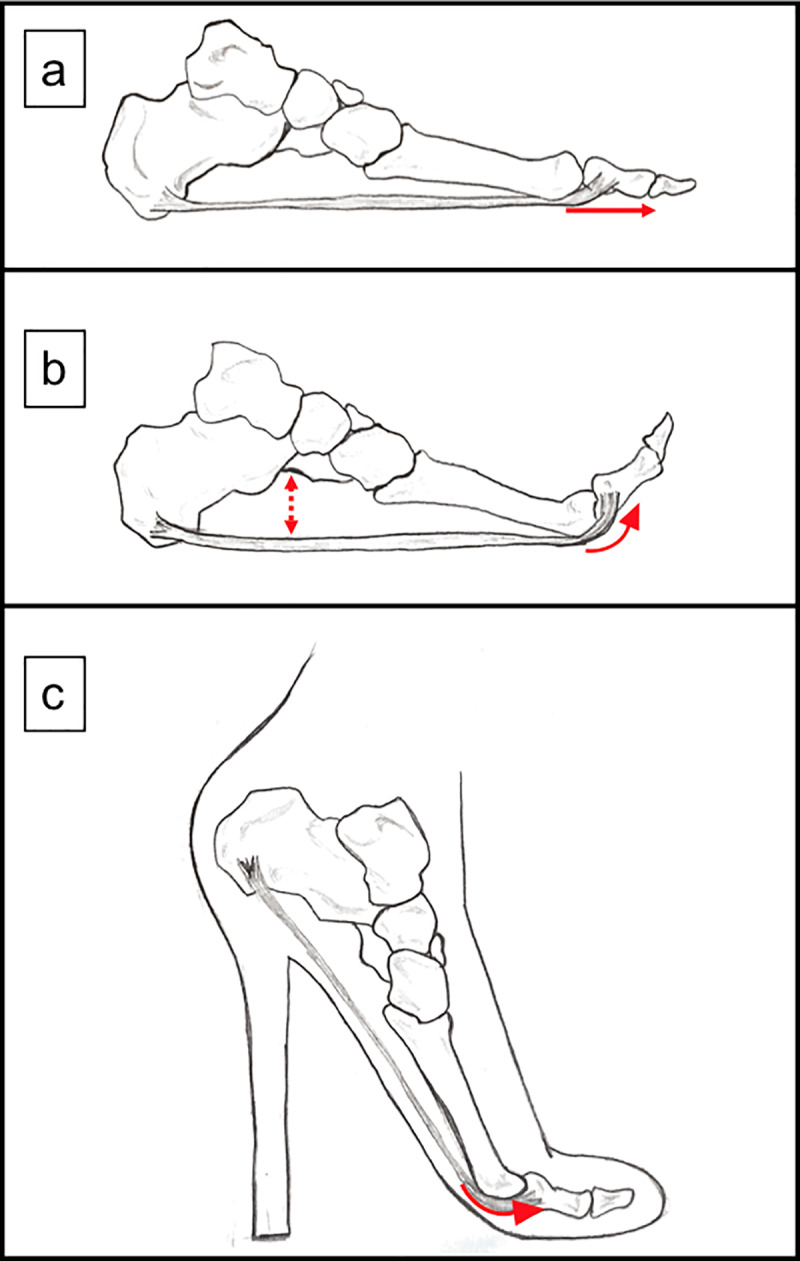
Windlass mechanism. a) the plantar fascia connects the calcaneus and the proximal metatarsophalangeal joint; b) when the proximal metatarsophalangeal joint is dorsiflexed, the fascia is tightened and the medial longitudinal arch raises; c) in HH, the proximal metatarsophalangeal joints are passively dorsiflexed. This position may cause Windlass mechanism which leads to more tension in the forefoot when wearing HH.

The TAH was not significantly changed from 0DI to 15DI and 30DI, but the TAH ratio had significantly increased in 30DI compared to 0DI. When loaded, the arch normally collapses as a response to loads [10] and rebounds like a spring reusing this energy to transmit the forces forwards [16]; however, in the case of HH, it seems that the transverse arch stiffens which does not allow it to do its shock absorption function. In this case, improper force transmission happens which could lead to pain and injuries in the forefoot. Also, when toes are dorsiflexed [17, 18] or when the first metatarsophalangeal joint is extended [19], Windlass mechanism is activated [17, 18] (Fig 5). This mechanism raises the medial longitudinal arch (MLA) [19] and collapses the lateral longitudinal arch [17]. It stiffens the plantar aponeurosis to make the foot more rigid as a compensation to allow an efficient ankle plantar flexion movement [17]; by helping the MLA to absorb and return energy in dynamic conditions [20]. This function may be altered when the metatarsophalangeal joint’s dorsiflexion angle is unchanged [20], as in when wearing HH where the tension on the plantar fascia is increased causing pain [7, 21]. We suppose that the transverse arch is affected by this mechanism and is also raised as the fascia tightens, pulling on the metatarsal heads. These TAH results contradict our hypothesis. A previous report shows that the transverse arch collapses in long-term use of HH [6]. Our data and the previous data suggest that, the transverse arch is elevated when wearing HH for a short duration, but it collapses as passive loading persists causing the structure to fatigue. We suppose that, had we taken the results for a longer duration, the TAH may have a different result after fatigue. Following the same pattern of the transverse arch, the MLA also collapses after that the plantar fascia and the intrinsic muscles of the foot fatigue after a long duration of wearing HH, resulting in injuries.

As for the heights of the metatarsal heads, the heights of 2MTH, 3MTH and 4MTH had significantly lowered in 30DI compared to 0DI. Lowered height of the metatarsal heads is caused by the increased pressure on them, and pressure is increased on the metatarsal heads in HH [8]. Decreased metatarsal heads height can also mean the shrinking and compression of the soft tissues underneath it, and the reshaping on the arch due to the shift of the MTH laterally (although insignificant, we noticed increase in the length between MS~LS, 2MTH~3MTH and 3MTH~4MTH in 15DI compared to 0DI, and between LS~2MTH in 30DI compared to 15DI), which may affect the height. The soft tissues under the metatarsal heads serve as cushions to protect the metatarsal heads from repetitive stresses [22, 23]. They also dissipate the energy under loading [8]. However, their function is lost, and the forces are not efficiently dispersed when under strain that accompanies the metatarsophalangeal joints’ dorsiflexion and the increased loading from the HH [8].

As for the SRA, it slightly lessened as HH increased, without significance. This may be due to the decreased length of the MS and the LS (as per our results) in response to loading. Previous reports are in agreement with the fact that the heights of the sesamoids decrease when loaded, noting that the MS bears more loads than the LS [24].

On the other hand, we had hypothesized that the transverse arch would widen. However, the TAL had shortened with significance in 15DI and in 30DI compared to 0DI, as well as the space between 4MTH and 5MTH in 30DI compared to 0DI. This may be due to the tightening of the deep transverse metatarsal ligaments, plantar ligaments, and dorsal interossei muscles that connect the metatarsal heads in the forefoot, and the dorsal interossei muscles and adductor halluces muscles; and the adduction of the metatarsals secondary to the Windlass mechanism [17]. In past studies, fatigue of these muscles was reported; however, in our study, the measurements took about five seconds to be complete which is not enough time for the ligaments and muscles to be fatigued. We suppose that these structures will elongate due to fatigue with time.

Furthermore, the total area under the metatarsal heads had significantly decreased in 15DI and in 30DI compared to 0DI. In details, the area between LS and 2MTH and the area between 4MTH and 5MTH has significantly decreased in 15DI compared to 0DI. This significant decrease in the area continues in 30DI between LS and 2MTH, 2MTH and 3MTH, 3MTH and 4MTH, and 4MTH and 5MTH compared to 0DI condition. This decrease in area is due to decreased height and length between the metatarsal heads, which are used to calculate the area in our study. Decreased area between the metatarsal heads and the plantar skin can cause tightening on the nerves, tendons and vessels located between the toes. This pattern is similar to Morton’s neuroma; a condition caused by repeated loading on the metatarsal heads causing trauma to the plantar intermetatarsal nerve and its impingement by the adjacent metatarsal heads and metatarsophalangeal joints [12, 21]. It is usually localized in the second and third intermetatarsal space [12] and it is documented to occur when wearing HH as the loads shift to the metatarsal heads [21]. We did not test whether our sample had Morton’s neuroma, but these results could be a clue to this painful condition. On the other hand, the lateral toes are known to be less stable than the medial toes [21], explaining the changes happening at the lateral level of the forefoot. What’s more, the lowered height of the metatarsal heads and decreased area underneath them reflects the tightening of the soft fatty pads under the metatarsal heads. Soft tissue under the foot serves as shock absorbents and thinning of these tissues increases the risk of inflammation and injuries under repetitive stress [22, 23]. This could be an explanation to the metatarsal pain resultant from wearing HH. As mentioned earlier, our measurements took seconds to be complete without fatigue of the plantar fascia. In longer hours of wearing HH, the plantar fascia may also collapse, and the soft tissues and the metatarsal heads will bear more pressure.

Women have more elastic joints by nature [25, 26] which puts them at higher risks of injuries and pain than men, and by wearing HH, this rate may increase. What’s more, in modern fashion, extraordinary heel heights exceeding 12cm (5 inch), or even ‘heelless heels” [27], are seen. Although there are not enough studies about the effect of such HH of the foot, women have already been advised not to wear HH higher than 5cm (about 2 inch) [28, 29]. To a certain extent, the effect of these extraordinary HH would be expected to be similar to the documented effects of HH, which worsen as the heel height increases. The documented effects are various, structural and morphological [4, 5, 28, 30–32]; such as: posture instability and irreversible postural deviations; increased compression pressure on the lumbar spine and activity of the erector spine muscle, leading to back pain; deformities of the toes and calluses under the metatarsal heads; with a remarkable worsening of overuse injuries and ankles injuries secondary to wearing HH. Furthermore, the frequency of wearing these heals will stiffen and fatigue the muscles (notably the tibialis anterior, lateral gastrocnemius and medial gastrocnemius [33]). It may be difficult to convince modern women to stop wearing HH, but it is not impossible to raise awareness about rotating shoe types (changing the type of shoes worn on daily basis) and avoiding their use at work as a dress code for companies, especially for jobs that require standing for long periods of time. Using the wrong footwear (footwear appropriate to the activity to be done as it helps with force and motor control [2]) and standing for long periods of time submit the foot to efforts that the feet are not made to bear and therefore causing damage [18].

Although this study describes the changes that happen at the level of the transverse arch in HH, it has several limitations. First, the duration of the measurements is short, and the shape of the arch may change when the muscles are fatigued after longer durations of wearing HH. Second, the custom-made HH are standardized to the average foot size in Asian women which may not have fit all our participants. Third, these custom-made heels had a wide heel and no toe box, and the effects may be different had it been a stiletto-heel type or a closed narrow toe box. Nonetheless, we have shown the detailed changes that happen to the transverse arch in HH. These results can be used for a better customization of HH or the paddings used with the HH.

## Conclusion

Short term standing in HH shortens the transverse arch and tightens the area between the metatarsal heads and the plantar skin of the foot. In this study, we showed that HH affected the shape of the transverse arch and these effects increased as the height of the heel increased.
